# Neonatal Diabetes: Case Report of a 9-Week-Old Presenting Diabetic Ketoacidosis Due to an Activating *ABCC8* Gene Mutation

**DOI:** 10.1177/2324709617698718

**Published:** 2017-03-24

**Authors:** Shawn Sood, Hannah Landreth, Jessee Bustinza, Laura Chalmers, Roopa Thukaram

**Affiliations:** 1 The University of Oklahoma, Tulsa, OK, USA; 2 The Children’s Hospital at Saint Francis, Tulsa, OK, USA

**Keywords:** neonatal diabetes mellitus, *ABCC8*, KATP, diabetic ketoacidosis, Kir6.2, KCNJ11, pediatric intensive care unit, sulfonylurea

## Abstract

**Context:** Neonatal diabetes mellitus, a rare condition occurring in approximately 1 in 500 000 live births, is defined as insulin-requiring hyperglycemia presenting in the first months of life. Neonatal diabetes can be transient or permanent, with studies characterizing the condition as a monogenic disorder. **Case Report:** We describe a case of a 9-week-old infant with neonatal diabetes who presented in diabetic ketoacidosis due to a mutation affecting the *ABCC8* gene that encodes the SUR1 subunit of the potassium ATP channel. **Conclusion:** This genetic diagnosis has therapeutic implications regarding the initiation of sulfonylurea administration as 85% of patients with neonatal diabetes due to *ABCC8* gene mutations can be successfully treated with oral sulfonylurea treatment.

## Introduction

Neonatal diabetes, a form of diabetes mellitus, is defined by persistent hyperglycemia in the first months of life.^1-5^ It can be further classified into transient neonatal diabetes, which remits early, or permanent neonatal diabetes, which requires lifelong therapy.^6^ The ATP-sensitive potassium channel, or KATP channel, is the central gatekeeper of electrical activity and thereby insulin secretion in the pancreatic beta cell.^3,4^ The most common cause of permanent neonatal diabetes mellitus is a heterozygous activating mutation in the KCNJ11 gene encoding Kir6.2.^7,8^ Kir6.2 and the sulfonylurea receptor, SUR1, comprise the pore forming and regulatory subunits of the KATP channel, respectively. SUR1 is encoded by the *ABCC8* gene and sulfonylureas bind directly to SUR1 to inhibit KATP channel activity.^8^

We report a case of a 9-week-old presenting in diabetic ketoacidosis and later diagnosed with an activating *ABCC8* mutation who is in the process of transitioning from insulin therapy to sulfonylurea.

## Case History

A 9-week-old female presented via air transport to our institution with a blood glucose of 1086 mg/dL and bicarbonate of less than 5 mmol/L reported at an outside emergency department. Concurrent with her hyperglycemia and acidosis, the patient presented with severe dehydration, respiratory distress, and lethargy. In the days prior to admission, she had decreased oral intake, increased lethargy, vomiting, diarrhea, and fever of 38°C. History was also significant for a weight loss of 1000 g in the 4 days prior to admission. Family history was pertinent for nonconsanguineous, healthy parents with paternal history of hypothyroidism but no other familial autoimmune diseases.

During air transport to our pediatric intensive care unit, the patient received two 20 mL/kg normal saline boluses and was placed on an insulin drip. On arrival, the patient was intubated secondary to respiratory distress, and a central line was placed to improve hemodynamic monitoring and allow administration of intravenous fluids and insulin. An initial venous blood gas at our institution revealed the following: pH 6.87, CO_2_ 26 mm Hg, base deficit 27.2 mmol/L, bicarbonate 4.7 mmol/L, lactate 2.7 mmol/L, sodium 154 mmol/L, potassium 5.3 mmol/L, and glucose 476 mg/dL. Urinalysis demonstrated 4+ sugars and 3+ ketones. Her β-hydroxybutyrate level was 8.10, indicating a diagnosis of diabetic ketoacidosis. Labs also revealed a white blood cell count of 17 600/cm and 12.0 band percentage. Given her history of fever and a high white blood cell count, a full septic workup was performed, and the patient was started on empiric broad spectrum antibiotics. Fluid resuscitation occurred over the next 48 hours ensuring steady adjustment of serum sodium and osmolarity. Acidosis and ketonuria resolved within 36 hours. Blood sugars were checked every 3 hours, and the patient was extubated 3 days after admission in a hemodynamically stable condition. Cultures from initial septic workup returned as negative, so antibiotics were discontinued based on newfound established etiology of her presentation.

The patient was transitioned from insulin drip to subcutaneous insulin and Humalog with correction factor for blood sugar greater than 150 mg/day, and 5 days status post hospital admission, the patient was discharged home. The patient is followed outpatient by our pediatric endocrinologist who chose to transition the patient from insulin to twice daily glyburide (Glibenclamide) therapy. Three months following her discharge, she continued to have excellent blood glucose control on glyburide 0.2 mg/kg divided twice daily.

Genetic testing results were ascertained after the patient was discharged and showed that the patient had a heterozygous pathogenic variant in *ABCC8*, specifically a variant located in exon 38 of *ABCC8* resulting in the substitution of methionine for valine at amino acid position 1522 of the protein. This variant, in the heterozygous state, has been previously reported to be a de novo, pathogenic variant in an individual with permanent neonatal diabetes and is consistent with an autosomal dominant form of neonatal diabetes mellitus.

## Discussion

Activating SUR1 mutations reduce sensitivity to the inhibitory actions of ATP and increase sensitivity to the stimulatory actions of ADP, which causes the KATP channel to remain open, even in the presence of glucose, thus preventing insulin release.^9,10^ Sulfonylureas act by an ATP-independent mechanism by binding to KATP channels, blocking them, to close these channels even when mutations are present and thereby mimicking the effect of glucose metabolism.^3,9,10^ This results in insulin release and is therefore a potential treatment option in neonatal diabetes caused by mutations in these subunits.^3,6,9^ Rafiq et al found that 85% of patients with neonatal diabetes due to *ABCC8* gene mutations could be successfully treated with oral sulfonylurea treatment and that lower doses of sulfonylureas were needed in SUR1 patients compared with the Kir6.2 patients.^9^

A wide variety of phenotypes is observed in patients with neonatal diabetes caused by *ABCC8* mutations: transient neonatal diabetes mellitus,^6,11^ permanent diabetes diagnosed before 6 months of age,^6,10,11^ permanent diabetes diagnosed in adolescence or later,^6,12,13^ or developmental delay, epilepsy, and neonatal diabetes, or DEND syndrome.^9^ Patch et al noted that in addition to neonatal diabetes, neurological features such as developmental delay, muscle weakness, and epilepsy were identified in 31% of probands with an *ABCC8* mutation.^12^ SUR1 is expressed in many other tissues, but the expression pattern of *ABCC8* restricts the pairing of the SUR1 protein subunits with Kir6.2 to pancreatic beta cells and the brain, thereby accounting for both neonatal diabetes and the neurological phenotype.^8,14^

The transfer from insulin to sulfonylurea treatment in SUR1 patients can be done either in the inpatient or outpatient setting. Rafiq et al suggest a gradual 0.1 mg/kg/day increase in glyburide dose during an inpatient setting.^9^ The time from the start of sulfonylurea therapy to eliminating exogenous insulin varies from 1 day to months.^3,14^ Vaxillaire et al^15^ noted that majority of SUR1 mutations are linked to transient neonatal diabetes, and it is yet to be determined whether our patient presents with transient or permanent neonatal diabetes, given that this case report was written 2 months after the patient’s admission; however, genetic testing of our patient was analogous to an individual with a previously reported de novo, pathogenic variant with permanent neonatal diabetes.

There are undeniable benefits of sulfonylurea treatment, including improved metabolic control, which was seen in KCNJ11 mutation carriers after successfully switching to sulfonylurea.^14,16-18^ A further advantage of the sulfonylurea treatment is the flexibility to respond with appropriate insulin secretion and the relatively mild side effects of sulfonylurea treatment.^11^ Apart from transitory diarrhea, abdominal discomfort and morning nausea, high-dose glyburide has reported to cause the development of tooth discoloration. However, the discoloration seems to be easily removed by routine cleaning.^9,16^

Based on the aforementioned specific action of sulfonylureas on the KATP channel, only those neonatal diabetes genotypes that interfere with the structure and/or expression of the KATP channel respond entirely to sulfonylurea therapy. Thus, the American Academy of Pediatrics recommends genetic testing of infants less than 6 months of age presenting with neonatal diabetes.^19^ In addition to the discussed benefits of sulfonylurea versus insulin therapy, cost-effectiveness of genetic testing for sulfonylurea responsive neonatal diabetes has also been under review. One theoretical cost analysis study showed that genetic testing for KCNJ11 and ABCC8 mutations may provide a cost benefit in the realm of $12 000 after 10 years, while taking into account costs of genetic testing, supervised sulfonylurea conversion costs, costs of medications, and projected harm from poorly controlled diabetes.^20^

As evidenced by the case report and the many emerging studies on the therapeutic and genetic screening, implications of neonatal diabetes, early recognition of neonatal diabetes followed by genetic testing and appropriate therapy will likely improve patient outcomes. Advances in genetic screening technology and cost-effectiveness will likely make this more possible in the future.
